# Non-covalent synthesis of supermicelles with complex architectures using spatially confined hydrogen-bonding interactions

**DOI:** 10.1038/ncomms9127

**Published:** 2015-09-04

**Authors:** Xiaoyu Li, Yang Gao, Charlotte E. Boott, Mitchell A. Winnik, Ian Manners

**Affiliations:** 1 School of Chemistry, University of Bristol, Bristol BS8 1TS, UK; 2 Department of Chemistry, University of Toronto, Toronto, Ontario M5S 3H6, Canada

## Abstract

Nature uses orthogonal interactions over different length scales to construct structures with hierarchical levels of order and provides an important source of inspiration for the creation of synthetic functional materials. Here, we report the programmed assembly of monodisperse cylindrical block comicelle building blocks with crystalline cores to create supermicelles using spatially confined hydrogen-bonding interactions. We also demonstrate that it is possible to further program the self-assembly of these synthetic building blocks into structures of increased complexity by combining hydrogen-bonding interactions with segment solvophobicity. The overall approach offers an efficient, non-covalent synthesis method for the solution-phase fabrication of a range of complex and potentially functional supermicelle architectures in which the crystallization, hydrogen-bonding and solvophobic interactions are combined in an orthogonal manner.

## Abstract (web summary)

Ubiquitous in nature, hierarchical architectures are less commonly achieved in synthetic functional materials. Here, the authors design and carefully assemble block copolymer micelles into complex supermicelles using hydrogen bonding in orthogonal combination with other non-covalent interactions.

## Graphical abstract

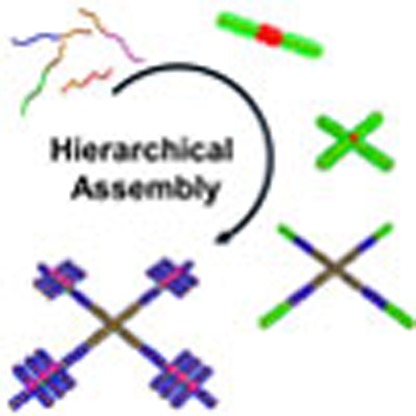

The intricate hierarchical structures achieved by nature^1^ using orthogonal interactions on different length scales have inspired the pursuit of artificial materials with complexity and functionality using analogous synthetic concepts^2^,^3^. Recent breakthroughs in colloidal particle synthesis have enabled their use as building blocks for higher-level assembly^4^. In particular, block copolymers provide a diverse array of soft-matter nanoparticles of varied shape and size^5^,^6^,^7^,^8^,^9^,^10^, and intricately designed structures such as Janus^11^,^12^,^13^,^14^,^15^ and patchy particles^16^,^17^,^18^,^19^. Several reports have described the assembly of these nanoparticles through electrostatic^20^ or solvophobic interactions^11^,^12^,^13^,^14^,^15^,^16^,^17^,^18^,^19^ into discrete multicomponent composite supermicelles^11^,^12^ or extended superstructures^13^,^14^,^15^,^16^,^17^,^18^,^19^.

Hydrogen-bonding (H-bonding) interactions, which play pivotal roles in the spontaneous organization of building blocks into hierarchical structures in natural systems, have been extensively used to assemble artificial structural units, as illustrated by work on small molecules^21^,^22^,^23^,^24^,^25^, DNA origami^26^,^27^,^28^, polymer/small molecule pairs^29^,^30^,^31^, polymer/polymer pairs^32^ and polymer/inorganic particles^33^. However, the creation of nanoscale architectures with similar levels of precision to nature by low-cost solution-phase processing remains a key challenge.

Here, we describe the use of cylindrical block comicelles with crystalline cores as building blocks to create complex hierarchical supermicelle structures via H-bonding interactions. Specifically, we demonstrate that fine control of the length and position of H-bonding donor and acceptor segments, made possible by the living crystallization-driven block copolymer self-assembly method, allows the interactions to be confined and the subsequent hierarchical organization of the resulting cylindrical block comicelles to be precisely directed.

## Results

### The basic polymer components and building blocks

**Figure 1:**
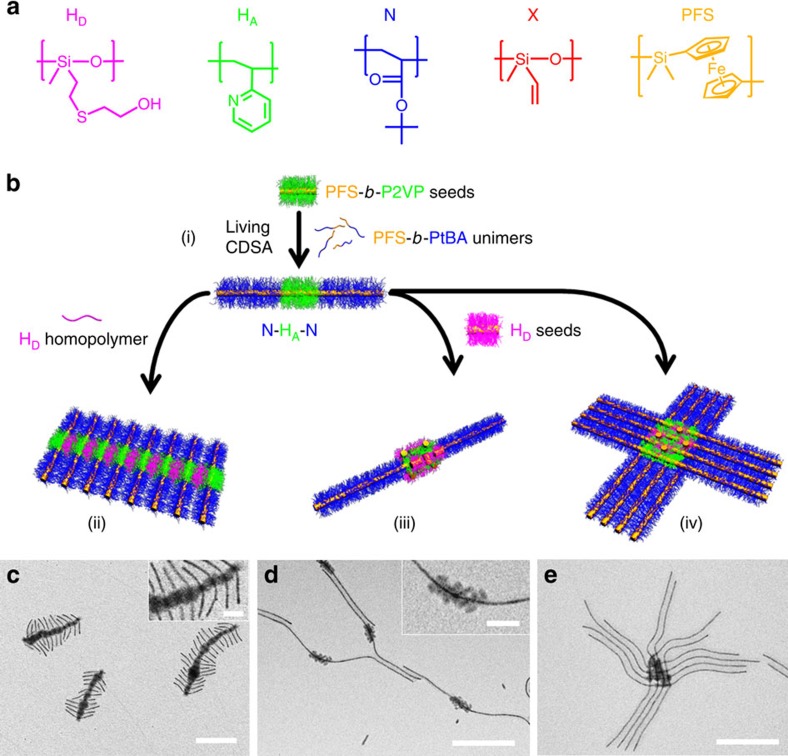
Initial studies of hierarchical assembly via H-bonding interactions. (**a**) Chemical structures of PMVSOH (H_D_), P2VP (H_A_), PtBA (N), PMVS (X) and PFS. (**b**) Schematic of the assembly processes. TEM images after solvent evaporation of supermicelles formed by (**c**) mixing H_D_ homopolymer (PMVSOH_105_) with N-H_A_-N (540 nm) triblock comicelles (hydroxyl/pyridyl group ratio=5:1); and H_D_ seeds (M(PFS_20_-*b*-PMVSOH_120_), 37 nm) with N-H_A_-N (1.4 μm) triblock comicelles (hydroxyl/pyridyl group ratio: (**d**) 20:1 and (**e**) 1:2) in *i*-PrOH. Insets in **c** and **d** are high magnification images of the H_A_ segments after the adsorption of H_D_ homopolymer and seeds, respectively. Scale bar, 500 or 100 nm (inset). Segments: H_A_=M(PFS_32_-*b*-P2VP_448_) and N=M(PFS_20_-*b*-PtBA_280_).

In Fig. 1a, we show the five different polymers that form the block copolymers studied. All of the block copolymers possessed a crystallizable, core-forming poly(ferrocenyldimethylsilane), PFS block together with a corona-forming segment that was either an H-bonding donor block (hydroxyl-functionalized poly(methylvinylsiloxane), PMVSOH, H_D_), an H-bonding acceptor block (poly(2-vinylpyridine), P2VP, H_A_), a non-interactive block (poly(*tert*-butyl acrylate), PtBA, N) or a crosslinkable block (poly(methylvinylsiloxane), PMVS, X). For convenience, as the crystalline PFS core was a common feature, all of the micelles are depicted in an abbreviated form that reflects their coronal chemistry (for example, triblock comicelle M(PFS_20_-*b*-PtBA_280_)-*b*-M(PFS_32_-*b*-P2VP_448_)-*b*-M(PFS_20_-*b*-PtBA_280_) is described as having segments of N, H_A_ and N and is written as N-H_A_-N). Cylindrical block comicelle building blocks such as N-H_A_-N were prepared via living crystallization-driven self-assembly (CDSA)^33^,^34^,^35^,^36^,^37^,^38^. In this process (see Fig. 1b (i)), dissolved block copolymer unimers with a crystallizable core-forming block and different corona-forming blocks can be added sequentially to short cylindrical micelles (seeds), which were prepared by sonication. This allowed the formation of monodisperse cylindrical micelles with segmented structures and lengths controlled from 50 nm to 5 μm (detailed characteristics of all seeds and triblock comicelles used in this study are shown in Supplementary Table 1).

### Initial studies of hierarchical assembly via H-bonding interactions

The H-bonding interactions between hydroxyl and pyridyl groups in polymers have been exploited extensively^29^,^30^,^31^,^32^,^39^, and our initial studies focused on exploring their potential use in supermicelle formation. We found that the addition of H_D_ homopolymer to block copolymer seeds with an H_A_ corona in isopropanol (*i*-PrOH, a selective solvent for both PMVSOH and P2VP blocks) led to the formation of an insoluble precipitate (Supplementary Fig. 2k). On the basis of this observation, we investigated the more controlled interactions between the H_D_ homopolymer and N-H_A_-N triblock comicelles, in which the H_A_ polymer chains were confined to the corona of the central segment of the cylindrical building block (Fig. 1b). From transmission electron microscopy (TEM) analysis, addition of H_D_ homopolymer to the *i*-PrOH solution of the triblock comicelles at a mole ratio of hydroxyl/pyridyl groups of 5:1 led to assembly of the comicelles through parallel aggregation of the central H_A_ coronal segments forming ‘fish-spine' supermicelles (Fig. 1b (ii); Supplementary Fig. 2g). The H_D_ homopolymer chains appeared to function as a ‘glue' to enable the triblock comicelles to assemble via H-bonding interactions. Although a distribution of very similar structures was formed in each case, substantial control could be imposed by adjusting various parameters. For example, use of either a very low or very high mole ratio of hydroxyl/pyridyl groups led to a significant reduction of the aggregation number (or arm distribution) in each supermicelle (Supplementary Fig. 2a–e). Furthermore, we were also able to tune the steric hindrance arising from the N segment by using several N segment-forming block polymers (PFS-*b*-PtBA) with different PtBA coronal block chain lengths. As shown in Supplementary Fig. 2f–j, with increasing PtBA block length and thus coronal spatial extent, the aggregation number also decreased.

Next, we replaced H_D_ homopolymer with block copolymer seeds bearing an H_D_ corona (Fig. 1b (iii) and (iv)) and studied how this changed the assembly behaviour. As an initial experiment, we mixed seeds with an H_D_ corona (*L*_*n*_=37 nm) and seeds with a corona of H_A_ (*L*_*n*_=45 nm) in *i*-PrOH. Seconds after mixing, a precipitate was observed in the solution (Supplementary Fig. 2l,m), which indicated the formation of large aggregates via uncontrolled H-bonding interactions. We then explored the analogous assembly process for the H_D_ seeds with N-H_A_-N triblock comicelles. At a ratio of hydroxyl/pyridyl groups of 20:1, TEM analysis revealed the formation of composite block comicelles with the H_D_ seeds attached to the surface of the central H_A_ segments. The presence of a small number of free H_D_ seeds was also detected (Fig. 1b (iii)). Interestingly, when the ratio was decreased to 1:2, instead of forming composite block comicelles, large micelle bundles were obtained, in which each H_D_ seed was bound to several N-H_A_-N triblock comicelles via H-bonding interactions (Fig. 1b (iv); Supplementary Fig. 2n). This behaviour contrasts to that observed in the case involving the addition of H_D_ homopolymer to N-H_A_-N triblock comicelles, which showed no aggregation at the same ratio (Supplementary Fig. 2a). We attribute the more extensive assembly to the larger size of H_D_ seeds compared with the homopolymer chains.

### Hierarchical assembly of triblock comicelles via H-bonding

**Figure 2:**
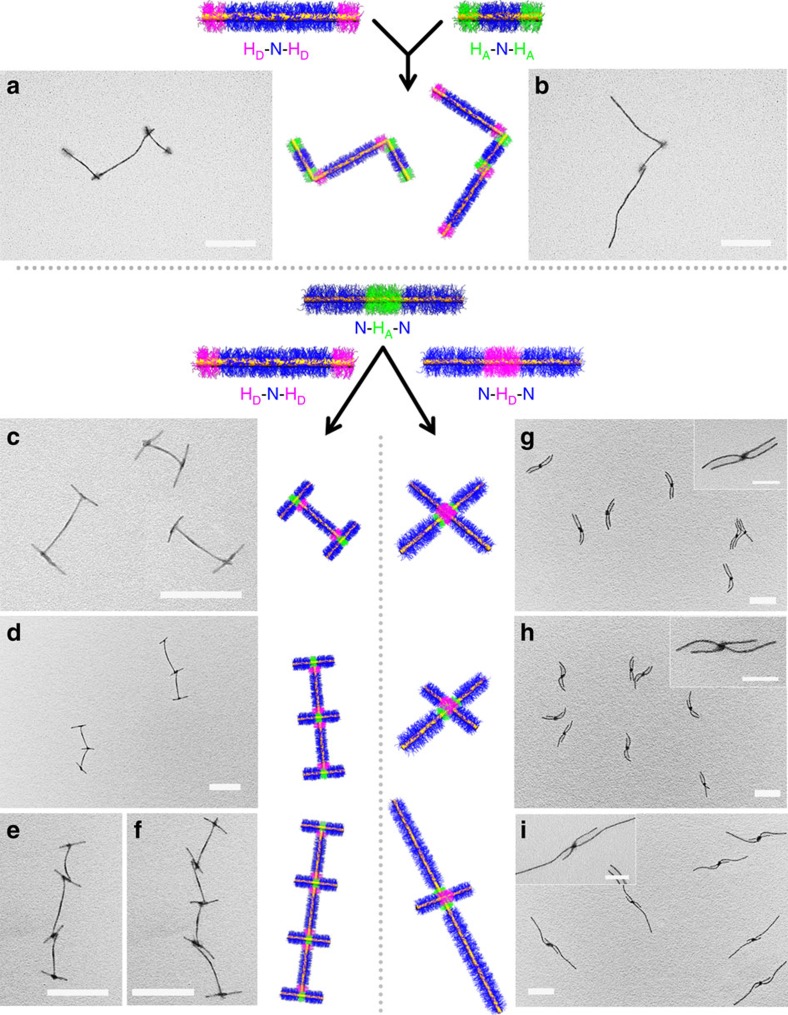
Hierarchical assembly of triblock comicelles via H-bonding. TEM images (after solvent evaporation) and corresponding schematic representations of (**a**) ‘ABA' and (**b**) ‘BAB' supermicelles from H_D_-N-H_D_ (910 nm) and H_A_-N-H_A_ (380 nm) triblock comicelles (hydroxyl/pyridyl groups of 3:1) in *i*-PrOH. (**c**–**f**) TEM images (after solvent evaporation) and corresponding schematic representations of **c**, ‘I'-shaped structures; **d**, dimers; **e**, trimers; **f**, tetramers from H_D_-N-H_D_ (480 nm) and N-H_A_-N (320 nm) triblock comicelles in *i*-PrOH (hydroxyl/pyridyl groups of 2:1). (**g**–**i**) TEM images (after solvent evaporation) of unsymmetrical micelles from N-H_D_-N and N-H_A_-N triblock comicelles in *i*-PrOH with total comicelle lengths of **g**, 540 and 540 nm; **h**, 330 and 540 nm; **i**, 330 nm and 1.3 μm, respectively. Scale bars are 500 nm and the scale bars in the insets are 200 nm. Segment H_D_=M(PFS_20_-*b*-PMVSOH_120_), H_A_=M(PFS_32_-*b*-P2VP_448_) and N=M(PFS_20_-*b*-PtBA_280_).

The studies described above suggested that the H-bonding interactions between hydroxyl groups on H_D_ chains and the pyridyl groups on H_A_ chains were sufficiently strong to build hierarchical structures with a higher level of complexity. We therefore investigated how confined H-bonding interactions would direct the assembly process for triblock comicelles. First, we explored a ‘condensation polymerization' of H_D_-N-H_D_ and H_A_-N-H_A_ triblock comicelles where the interacting H_D_ and H_A_ segments were both placed at the termini and were therefore only shielded by N segments on one side. To distinguish the two kinds of triblock comicelles in the resulting supermicelles, different N central segment lengths were used (680 and 240 nm for H_D_-N-H_D_ and H_A_-N-H_A_, respectively. See Supplementary Fig. 1 and Supplementary Table 1). Figure 2a,b show TEM images of the ‘ABA' and ‘BAB' supermicelles using a hydroxyl/pyridyl group ratio of 3:1. However, the intermicellar H-bonding interactions were not fully controlled as ‘branched' supermicelles were also detected (Supplementary Fig. 3a,b).

To achieve more effective confinement for the H-bonding interactions, the H_A_ segments were placed at the centre of a triblock comicelle. Subsequent mixing of H_D_-N-H_D_ and N-H_A_-N triblock comicelles (hydroxyl/pyridyl group ratio of 3:1) in *i*-PrOH led to ‘I'-shaped supermicelles (Fig. 2c). The supermicelles could be extended in length to form dimer, trimer, tetramer and even ‘polymeric' supermicelles (Fig. 2d–f; Supplementary Fig. 3c), but no branched structures were observed. When the N-H_A_-N triblock comicelles were in slight excess, predominantly ‘I'-shaped supermicelles were produced (Supplementary Fig. 3d).

When the H_D_ and H_A_ segments were both placed in the centre of the triblock comicelles and the resulting N-H_D_-N and N-H_A_-N cylindrical comicelles mixed in *i*-PrOH (hydroxyl/pyridyl group ratio of 2:1) ‘cross' supermicelles were formed (Fig. 2g). Unsymmetrical examples were prepared using N-H_D_-N and N-H_A_-N triblock comicelles with different lengths with high efficiency (yield=80%; Fig. 2h,i). This indicated that the balance between the attractive H-bonding between the H_D_ and H_A_ central segments and the repulsive, steric interactions of the coronas of the terminal N segments led only to one-to-one complexes. However, when this balance was disturbed, no ‘cross' supermicelles were formed. For example, compared with the samples shown in Fig. 2g–i, when the P2VP coronal block for the central H_A_ segment was reduced in length (from 448 to 250 repeat units) the weakened attraction between H_D_ and H_A_ segments was insufficient to generate ‘cross' supermicelles (Supplementary Fig. 3e). In contrast, multiple-to-one complex ‘cross' supermicelles were formed when an increased H_A_ segment length was used (109 nm, larger than the value of *L*_*n*_=45 nm in the cases of Fig. 2g–i) in the N-H_A_-N triblock comicelles (Supplementary Fig. 3f).

### Programmed stepwise hierarchical assembly

**Figure 3:**
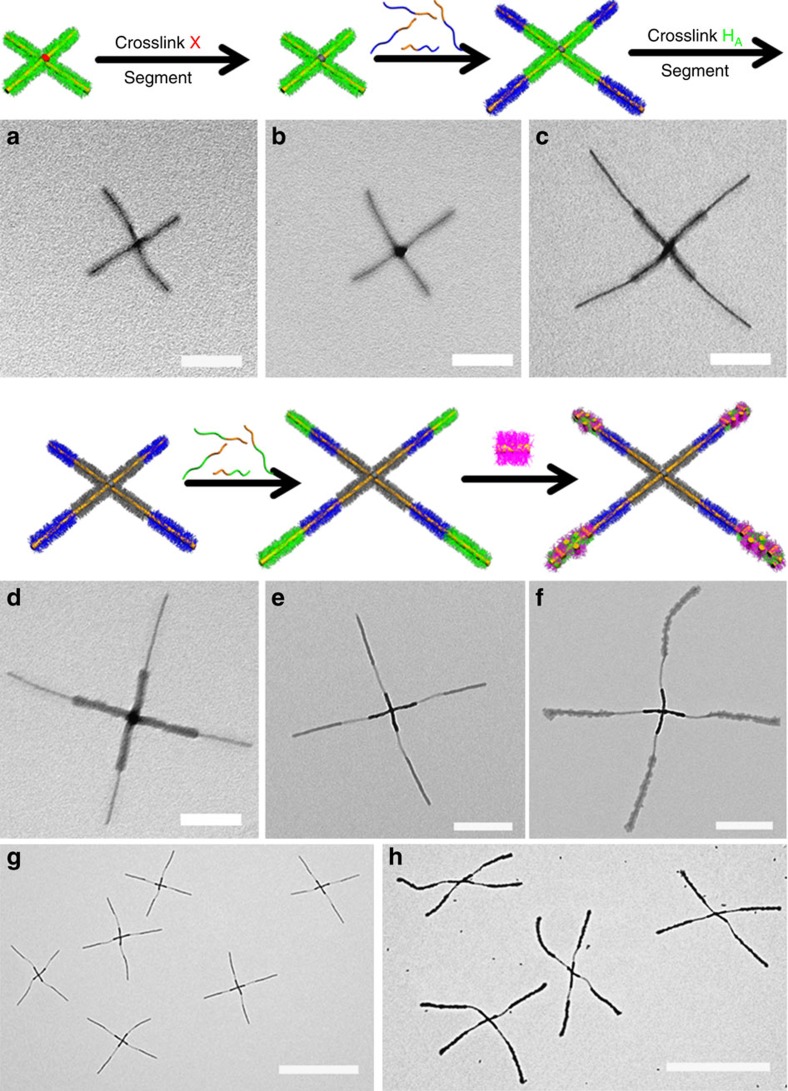
Programmed stepwise hierarchical assembly. TEM images (after solvent evaporation) and corresponding schematic representations of (**a**) ‘cross' supermicelles from the H_A_-X-H_A_ (420 nm) triblock comicelles; and the ‘cross' supermicelles (**b**) with crosslinked ^XL^X segments; (**c**) after the growth of N segments; (**d**) with crosslinked ^XL^H_A_ segments; (**e**) after the growth of another H_A_ segments; and (**f**) after the further addition of H_D_ seed micelles in *i*-PrOH. Scale bars are 200 nm in images **a**–**d**, 500 nm in images (**e**,**f**) and 2 μm in lower magnification images (**g**,**h**). Segment X=M(PFS_36_-*b*-PMVS_324_), H_A_=M(PFS_25_-*b*-P2VP_250_), N=M(PFS_20_-*b*-PtBA_280_) and H_D_=M(PFS_20_-*b*-PMVSOH_120_).

**Figure 4:**
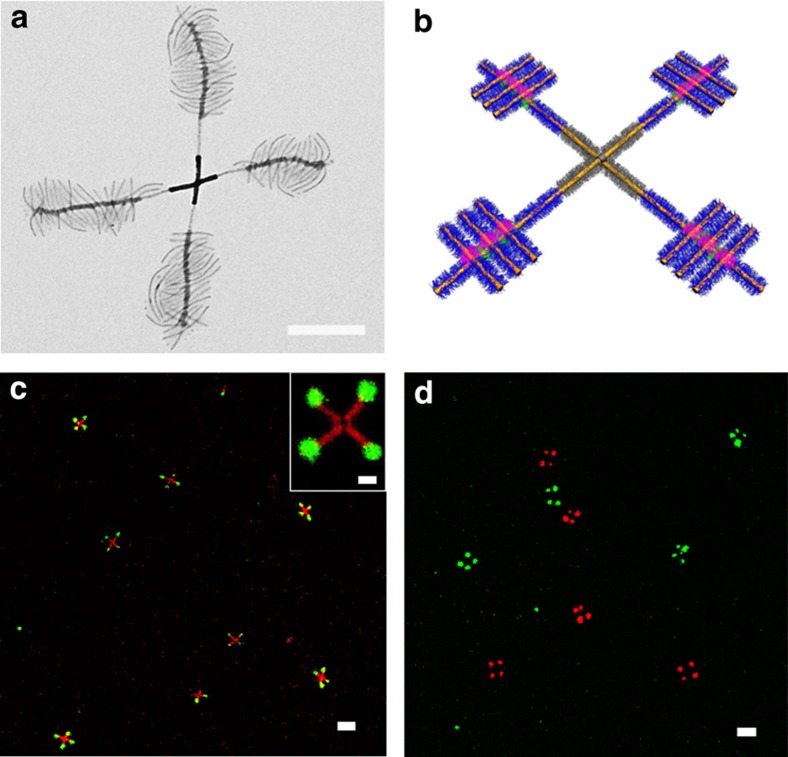
‘Windmill'-like supermicelles. (**a**) TEM image (after solvent evaporation) of a ‘windmill'-like supermicelle formed in *i*-PrOH. (**b**) Schematic illustration of the supermicelles. (**c**) LSCM image of fluorescent dye-labelled large ‘supermicelles' with the inner N segments labelled with a red dye and the outer N segments labelled with a green dye in *i*-PrOH. Inset is a zoom-in LSCM image of a ‘windmill'-like supermicelle. (**d**) LSCM images of the mixture of ‘windmill'-shaped supermicelles labelled with either red or green dyes (after 10 days at 22 °C) in *i*-PrOH. Scale bars are 500 nm in (**a**), 5 μm in (**c**,**d**) and 1 μm in the inset.

We also explored the use of a combination of H-bonding and solvophobic interactions to build even more complex, higher-level micelle architectures. First, H_A_-X-H_A_ triblock comicelles with crosslinkable central segments were prepared by living CDSA. After switching to a more polar solvent via dialysis (from *i*-PrOH/*n*-hexane=4:1 (v/v), to MeOH), these triblock comicelles formed ‘cross' supermicelles, due to the aggregation of X segments via solvophobic interactions (Fig. 3a). The yield of ‘cross' supermicelles was *ca.* 90% (Supplementary Fig. 4a). The X segments were then subject to covalent crosslinking^40^ using UV irradiation to create ^XL^X regions to make the ‘cross' supermicelles permanent (Fig. 3b; Supplementary Fig. 4b). To build the next level of structure, N segments were grown from the four termini of these supermicelles by living CDSA (Fig. 3c; Supplementary Fig. 4c). To impart further robustness to the supermicelles and to hinder the acceptor ability of the P2VP chains during subsequent transformations, the H_A_ coronal segments from the initial H_A_-X-H_A_ triblock comicelle were crosslinked using Pt nanoparticles^13^ to yield ^XL^H_A_ segments (Fig. 3d; Supplementary Fig. 4d). As a result of the crosslinking, the electron density of ^XL^H_A_ and ^XL^X segments increased and they appeared darker than the other regions by TEM. A further H_A_ segment was then created at each of the cross supermicelle termini by living CDSA (Fig. 3e), and seeds with an H_D_ corona were then added to bind to the uncrosslinked H_A_ segments via H-bonding (Fig. 3f). The resulting composite ‘cross' supermicelles were uniform in size and architecture, demonstrating the precision of this controlled hierarchical assembly method (see low resolution TEM images in Fig. 3g,h). To further illustrate the level of control, we grew H_A_ segments with a longer P2VP corona-forming block at the termini of ‘cross' supermicelles and we were then able to bind more H_D_ seeds and to form larger bundles on the arms of composite ‘cross' supermicelles (Supplementary Fig. 5a–c). Moreover, the bound H_D_ seeds were found to be still active to living CDSA^33^ and addition of the PFS-*b*-PtBA unimer led to the formation of N segments (Fig. 4a). Interestingly, the subsequent growth of the N segments led to their mutual repulsion and resulted in a perpendicular alignment of the N-H_D_-N comicelles relative to the cross supermicelle arms. This afforded hierarchical ‘windmill-like' supermicelles with high uniformity in size, architecture and yield (Fig. 4a,b; Supplementary Fig. 4e).

Direct characterization of the supermicelles in solution was achieved using laser scanning confocal microscopy (LSCM) analysis of ‘windmill'-shaped supermicelle analogues with N segments labelled with red or green fluorescent BODIPY dyes. ‘Cross' micelles with red-dye-labelled N segments in the arms were prepared first (Supplementary Fig. 5d–g). Finally, green-dye-labelled N segments were grown from the H_D_ seeds by living CDSA (Fig. 4c). A key issue for supramolecular, H-bonded assemblies is their potential for dynamic behaviour. To explore the possible lability of the perpendicularly arranged N-H_D_-N comicelles near the termini of the ‘cross' supermicelle arms, we mixed supermicelles in which the corresponding N segments possessed green dyes with analogues containing only red dyes in *i*-PrOH. No significant exchange between the two kinds of ‘windmill' supermicelles was detected by LSCM even after 10 days at 22 °C, which indicated that they are static under these conditions and exist as kinetically stable, non-equilibrium structures (Fig. 4d; Supplementary Fig. 6g–j; for control experiments see Supplementary Fig. 6a–f).

## Discussion

In summary, these results demonstrate the programmed formation of complex supermicelles by the use of confined regions of hydrogen bonding in cylindrical micelle precursors in orthogonal combination with crystallization and solvophobic interactions. This non-covalent synthesis approach allows the high-yield formation of hierarchical architectures that are persistent in solution, kinetically stable, easy to functionalise and sufficiently robust to allow for further processing. Additional stabilization can also be introduced through covalent crosslinking methods. The novel architectures accessible may be useful as models of biological structures (for example, chromosomes in the case of unsymmetrical cross-micelles) or as tectons for the creation of yet more complex hierarchical materials. As the living CDSA method used to create the micelle-building blocks has been shown to be applicable to a variety of crystallizable block copolymers and related π-stacking molecular amphiphiles, including those based on electroactive^41^,^42^,^43^,^44^,^45^, bioactive^46^,^47^ and bioinert^48^ characteristics, potential future applications for the resulting assemblies in sensing, nanoelectronics, catalysis and biomedicine can be envisaged.

## Methods

### Polymer synthesis

The diblock copolymers used in this study were synthesized via anionic polymerization in an inert atmosphere glovebox. The detailed polymerization procedures for these polymers have been reported elsewhere^49^,^50^,^51^. The PFS-*b*-PMVSOH polymer was prepared from PFS-*b*-PMVS polymer via a thiol-ene click reaction (with 2-mercaptoethanol), which has been reported recently by our group^52^.

Fluorescent dye-labelled PFS-*b*-PtBA was obtained by post-polymerization modification. The PFS-*b*-PtBA precursor with a hydroxyl terminal group was synthesized via anionic polymerization and the anions were quenched with ethylene oxide and subsequently with 4-*tert*-butylphenol (yield 99% by ^1^H NMR). Green dye (BODIPY FL, 3 mg, 8 × 10^−3^ mmol of carboxyl groups)^38^ was mixed with 80 mg of the PFS-*b*-PtBA with a hydroxyl terminal group (2 × 10^−3^ mmol of hydroxyl groups), 1 mg of dicyclohexylcarbodiimide (8 × 10^−3^ mmol) and 0.8 mg of 4-dimethylaminopyridine (1 × 10^−3^ mmol) in dry tetrahydrofuran (THF) (2 ml)^38^. The solution was stirred at room temperature for 3 days before the polymer was precipitated into a mixed solvent of MeOH and water (8:2, v/v) for three times and further by dialysis against THF. The red dye-containing polymer was prepared the same way except BODIPY 630/650 was used^38^.

### Polymer characterization

Gel permeation chromatography (GPC) measurements were carried out on a Viscotek VE 2001 triple-detector gel permeation chromatograph equipped with an automatic sampler, a pump, an injector, an inline degasser and a column oven (30 °C). The elution columns consist of styrene/divinyl benzene gels with pore sizes of 500 and 100,000 Å. Detection was conducted by means of a VE 3580 refractometer, a four-capillary differential viscometer, and 90° and low angle (7°) laser light (*λ*_0_=670 nm) scattering detectors, VE 3210 and VE 270. THF (Fisher) was used as the eluent, with a flow rate of 1.0 ml min^−1^. Samples were dissolved in the eluent (2 mg ml^−1^) and filtered with a Ministart SRP 15 filter (polytetrafluoroethylene membrane of 0.45 μm pore size) before analysis. The calibration was conducted using a PolyCALTM polystyrene standard from Viscotek. To determine the molar mass of the block copolymers, aliquots of the first block were taken and the molar mass of the first block was determined by gel permeation chromatography. The polymerization degrees of the two blocks were then determined by combining the molecular weight *M*_*n*_ of the first block with the block ratio of the diblock copolymer, which was obtained by integration of the ^1^H NMR spectrum. Polydispersities of the PFS homopolymers and block copolymers were in the range 1.08–1.16 by GPC.

### Transmission electron microscopy

Copper grids from Agar Scientific, mesh 400, were coated with a carbon film. Carbon coating was done using an Agar TEM Turbo Carbon Coater in which carbon was sputtered onto mica sheets before deposition on the grids via flotation on water. The samples for electron microscopy were prepared by drop-casting one drop (∼10 μl) of the micelle colloidal solution onto a carbon-coated copper grid. Bright-field TEM micrographs were obtained on a JEOL 1200-EX II microscope operating at 120 kV and equipped with an SIS MegaView III digital camera. Images were analysed using the ImageJ software package developed at the US National Institutes of Health. As the micelles reported here are trapped kinetically because of crystallization of the core block, the morphologies observed from dried samples by TEM are anticipated to match closely those observed in solution, as previously shown for 1D systems^36^. For the statistical length analysis, ∼300–400 objects were measured to determine the contour length. Histograms of the length distribution were constructed. From these data, values of the s.d. of the length distribution ‘sigma' were determined. The number average micelle length (*L*_*n*_) and weight average micelle length (*L*_*w*_) were calculated using the equations (1) and (2) below from measurements of the contour lengths (*L*_*i*_) of individual micelles, where *N*_*i*_ is the number of micelles of length *L*_*i*_, and *n* is the number of micelles examined in each sample.


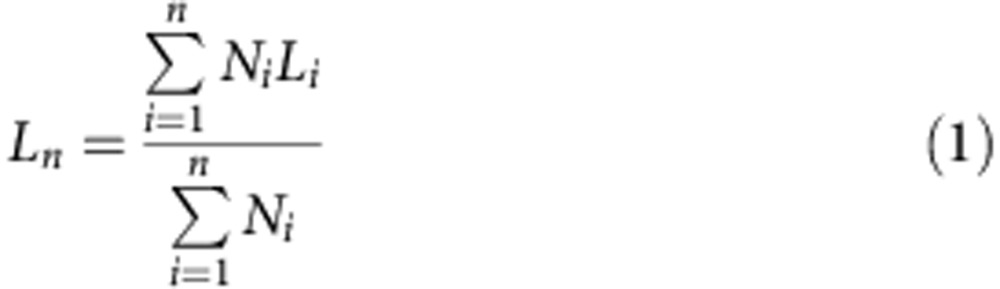



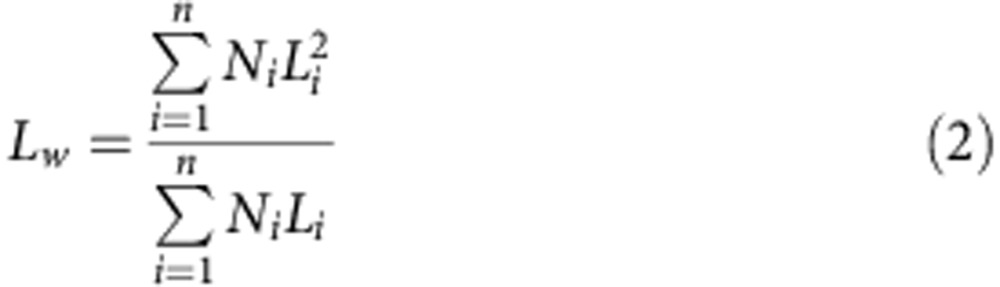


The distribution of micelle lengths is characterized by both *L*_*w*_/*L*_*n*_ and the ratio σ/*L*_*n*_, where σ is the s.d. of the length distribution.

### Experimental evidence for H-bonding interactions

PMVSOH_192_ and P2VP_20_ homopolymers were dried from their *i*-PrOH solution and dried in vacuum oven before characterization. The mixture of PMVSOH and P2VP (mole ratio of hydroxyl/pyridyl group=1:1) was prepared by mixing the two solutions and dried before characterization.

Fourier Transform Infrared (FTIR) spectra were recorded with an attenuated total reflection accessory. As can be seen from Supplementary Fig. 2o, a new peak appeared at 3,264 cm^−1^ after mixing the two homopolymers suggesting the formation of H-bonds^53^,^54^. The peak at 3,390 cm^−1^ originated from the residual *i*-PrOH molecules and the homopolymers (O-H stretching of H-bonded and free hydroxyl groups). The peaks at 1,565 and 1,580 cm^−1^ (from P2VP homopolymer, as shown in Supplementary Fig. 2p), corresponding to the stretching of double bonds in the pyridine ring, shifted to 1,631 and 1,658 cm^−1^ after mixing with PMVSOH homopolymers, a typical response to hydrogen bonding of pyridine ring^54^,^55^.

### Preparation of seed cylinders

Seed cylinders were prepared by ultrasonicating (50 W sonication processor equipped with a titanium sonotrode) the block copolymer cylinders at low temperatures. PFS-*b*-PMVS seed cylinders were obtained via sonication at −78 °C for 1 h in *n*-hexane, and sonication at 0 °C for 1 h in *i*-PrOH was used to obtain other seed cylinders (M(PFS-*b*-P2VP) and M(PFS-*b*-PMVSOH)). The original cylinders were obtained by directly dispersing the diblock copolymers into the corresponding solvents and heating at 70 °C for 1 h before the solution was allowed to cool to room temperature. The lengths of seed cylinders could be increased by adding a solution of fresh unimers (the polymer dissolved in THF) into the seed micelle solution.

### Preparation of triblock comicelles

The triblock comicelles were prepared via the seeded growth method. For example, to prepare triblock comicelle M(PFS_20_-*b*-PtBA_280_)-*b*-M(PFS_32_-*b*-P2VP_448_)-*b*-M(PFS_20_-*b*-PtBA_280_), 0.1 ml of M(PFS_32_-*b*-P2VP_448_) seed cylinders *i*-PrOH solution (0.5 mg ml^−1^) was diluted with 0.4 ml of *i*-PrOH, and then 5 μl of a THF solution of PFS_20_-*b*-PtBA_280_ unimers was added. The PFS_20_-*b*-PtBA_280_ unimers then grew epitaxially from the active termini of the M(PFS_32_-*b*-P2VP_448_) seed cylinders to form triblock comicelles.

### Preparation of supermicelles via H-bonding interactions

Two kinds of cylinders, one bearing M(PFS-*b*-P2VP) segments and the other bearing M(PFS-*b*-PMVSOH) segments were mixed with each other at the desired feed ratio in *i*-PrOH. The solution was gently shaken for 20 s and kept still overnight before analysis by TEM.

The resulting supermicelles (such as those shown in Fig. 1c, the large micelle bundles in Fig. 1e and the linear ‘oligomers' in Fig. 2c–f) were reproducibly formed as a distribution of very closely related structures (for example, see low resolution TEM images in Supplementary Figs 2c,n and 3). For example, in the case of the micelle bundles, these were relatively uniform in size but the aggregation number (that is, arm distribution) generally varied between 5 and 10.

The yields of the simple symmetrical ‘cross' micelles and unsymmetrical ‘cross' micelles (Fig. 2) by TEM were *ca.* 80 and 90%, respectively.

### Preparation of complex ‘cross' and ‘windmill' supermicelles

The first level ‘cross' supermicelles prepared from H_A_-N-H_A_ triblock comicelle (M(PFS_25_-*b*-P2VP_250_)-*b*-M(PFS_36_-*b*-PMVS_324_)-*b*-M(PFS_25_-*b*-P2VP_250_)), which were initially prepared in a mixed solvent consisting of *n*-hexane/*i*-PrOH=1:4 (v/v) by the seeded growth method. The solvent was slowly switched to MeOH by changing the solvent stepwise (100% of *i*-PrOH, *i*-PrOH/MeOH=8:2, 6:4, 4:6, 2:8 and eventually 100% of MeOH). This slow solvent switching protocol was designed to gradually induce the aggregation of insoluble block and thus the formation of ‘cross' supermicelles. By counting from TEM images, *ca.* 80% of the objects observed from TEM are ‘cross' supermicelles. Most of (*ca.* 90%) the H_A_-N-H_A_ triblock comicelles formed ‘cross' supermicelles, and the others either remained as individual cylindrical comicelles in the solution or formed larger aggregates consisting of multiple triblock comicelles.

The MeOH solution of the ‘cross' supermicelles (1.0 ml) was degassed by N_2_ bubbling for 30 min before being transferred into a glovebox, where 10 μl of 2,2-dimethoxy-2-phenylacetophenone in MeOH (10 mg/ml) was added. The solution was subsequently subjected to UV irradiation for 30 min with occasional agitation. The collapsed and densely packed PMVS chains were crosslinked on UV irradiation.

After crosslinking of the PMVS block, the sample solution was dialyzed against *i*-PrOH to remove MeOH. After crosslinking, the ‘cross' supermicelles were stable in *i*-PrOH (Supplementary Fig. 4b). M(PFS_20_-*b*-PtBA_280_) segments were grown via seeded growth by adding THF solution of PFS_20_-*b*-PtBA_280_ unimers into the solution. The M(PFS_25_-*b*-P2VP_250_) segments were further crosslinked by Karstedt's catalyst. Typically, a mass ratio of 1:2 (pyridyl to Pt) was used. Subsequently, another M(PFS_25_-*b*-P2VP_250_) segment was grown onto the open termini of the M(PFS_20_-*b*-PtBA_280_) segments via seeded growth, after which M(PFS_20_-*b*-PMVSOH_120_) seeds were added into the solution with a ratio of 25:1 (hydroxyl/pyridyl). The M(PFS_20_-*b*-PMVSOH_120_) seeds adsorbed onto the uncrosslinked M(PFS_25_-*b*-P2VP_250_) segments due to H-bonding interactions. Into the solution fresh PFS_20_-*b*-PtBA_280_ was added again, which grew from the open termini, and finally ‘windmill'-shaped supermicelles were obtained.

As a result of the efficiency of living CDSA, the ‘cross' supermicelles were successfully and quantitatively converted into ‘windmill' supermicelles based on TEM images. Thus, about 90% of the supermicelles were ‘windmill' supermicelles. However, to avoid having the H_D_ seeds interact with multiple composite ‘cross' supermicelles, a slight excess of H_D_ seeds was used (Fig. 3f,h). The H_D_ seeds that did not become attached to the composite ‘cross' supermicelles were also able to initiate the growth of N segments and form N-H_D_-N triblock comicelles once PFS-*b*-PtBA unimers were added into the solution (Supplementary Fig. 4e, marked with red circles). Thus, by counting from TEM images, *ca.* 75% of the objects observed from TEM were ‘windmill' supermicelles. By counting the individual N-H_D_-N triblock comicelles and those present on the ‘windmill' supermicelles from TEM images, we estimate that *ca.* 95% of the N-H_D_-N triblock comicelles present were incorporated into the ‘windmill' supermicelles.

### Preparation of fluorescent ‘windmill' supermicelles

The fluorescent ‘windmill' supermicelles were prepared in the same way as the normal ones, except fluorescent dye-containing PFS_20_-*b*-PtBA_280_ unimers were used. Specifically, after the crosslinking of the H_A_ segments, as shown in Fig. 3d, dye-terminated PFS_20_-*b*-PtBA_280_-red unimers were added to extend the PFS-*b*-PtBA arms. Subsequently, PFS_25_-b-P2VP_250_ unimers were added to further extend the arms, after which M(PFS_20_-*b*-PMVSOH_120_) seeds were added into the solution with a ratio of 25:1 (hydroxyl/pyridyl). The M(PFS_20_-*b*-PMVSOH_120_) seeds adsorbed onto the uncrosslinked M(PFS_25_-*b*-P2VP_250_) segments. Finally, dye-terminated PFS_20_-*b*-PtBA_280_ unimers were added into the solution to generate the final ‘windmill' supermicelles.

### General techniques

Photolysis experiments were carried out with Pyrex-glass filtered emission from a water-cooled, 125-W medium-pressure mercury lamp (Photochemical Reactors Ltd.), emitting predominantly 365 nm. The emission lines of the mercury lamp were 577–579, 546, 436, 408–405, 366–365, 334, 313, 302, 297, 289, 280, 270, 265 and 254 nm. An ethylene glycol/deionized water bath in conjunction with a thermostat was used to maintain constant temperatures during photoirradiation. All the photoreactions were carried out at 20 °C.

All the ^1^H NMR characterization was carried out on a Varian 400 MHz instrument, with chemical shifts referenced to tetramethylsilane in *d*-chloroform (CDCl_3_). FTIR spectra were recorded with Cary 630 FTIR spectrometer fitted with an attenuated total reflection accessory (Agilent Technologies).

### Confocal fluorescence microscopy

Confocal imaging was performed using a Leica SP5 system attached to a Leica DMI6000 inverted epifluorescence microscope with a × 63 (NA 1.4) oil immersion objective lens. Fluorophores from PFS-*b*-PtBA-red and PFS-*b*-PtBA-green were excited using a HeNe laser operating at 594 nm and an argon laser operating at 488 nm, respectively. Confocal images were obtained using digital detectors with observation windows of 605–700 nm for the red dye and 500–570 nm for the green dye. The resulting outputs were obtained as digital false-colour images, which are colour coded using the chromaticity of each micelle block under UV irradiation at 365 nm, as observed by fluorescence spectroscopy. For imaging of multicomponent supermicelles, the output power of each laser was varied until the fluorescence of all blocks could be observed at approximately equal brightness.

## Additional information

**How to cite this article:** Li, X. *et al*. Non-covalent synthesis of supermicelles with complex architectures using spatially confined hydrogen-bonding interactions. *Nat. Commun.* 6:8127 doi: 10.1038/ncomms9127 (2015).

## Supplementary Material

Supplementary Information Supplementary Figures 1-6 and Supplementary Table 1
