# Supplementary material for: Non-covalent synthesis of supermicelles with complex architectures using spatially confined hydrogen-bonding interactions
Source: Nat Commun. 2015 Sep 4;6:8127. doi: 10.1038/ncomms9127 (PMC4569713; doi:10.1038/ncomms9127)
Supplement: Supplementary Information — Supplementary Figures 1-6 and Supplementary Table 1 [file ncomms9127-s1.pdf]

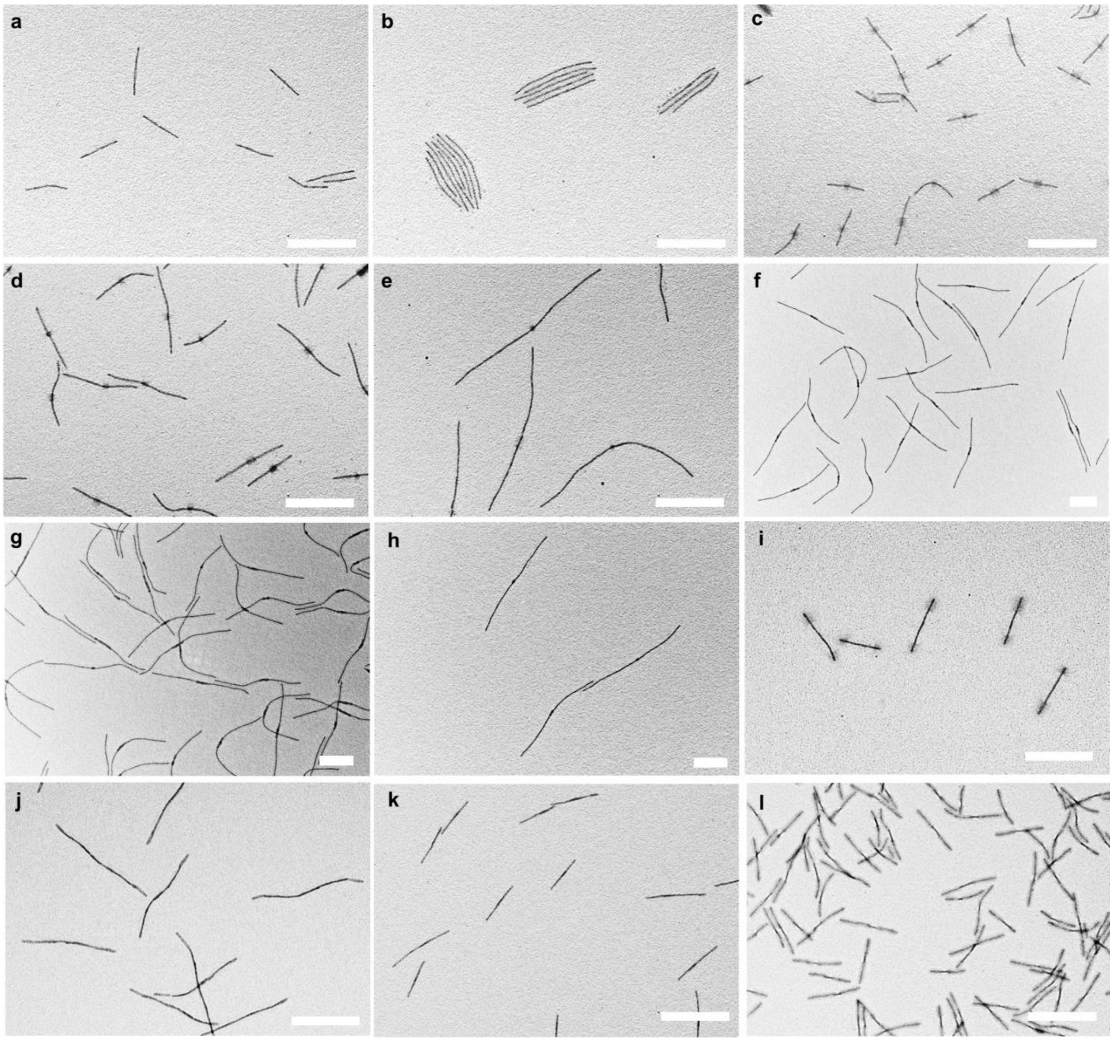

Supplementary Figure 1| Supporting images for triblock comicelle building blocks. TEM images (after solvent evaporation) of triblock comicelles **a**, N-H_D-N (330 nm); **b**, N-H_D-N (540 nm); **c**, N-H_A-N (320 nm); **d**, N-H_A-N (540 nm); **e**, N-H_A-N (1.3 μm); **f**, N-H_A-N (1.5 μm, middle H_A segment length = 109 nm); **g**, N-H_A-N (1.4 μm); **h**, N-H_A-N (1.6 μm); **i**, H_A-N-H_A (380 nm); **j**, H_D-N-H_D (910 nm); **k**, H_D-N-H_D (480 nm); and **l**, H_A-X-H_A (420 nm) in *i*-PrOH. Scale bars are 500 nm. Segment H_D = M(PFS_{20}-*b*-$PMVSOH_{120}$), N = M(PFS_{20}-*b*-$PtBA_{280}$), H_A = M(PFS_{32}-*b*-$P2VP_{448}$) except that in **h**, H_A = M(PFS_{25}-*b*-$P2VP_{250}$), X = M(PFS_{36}-*b*-$PMVS_{324}$).

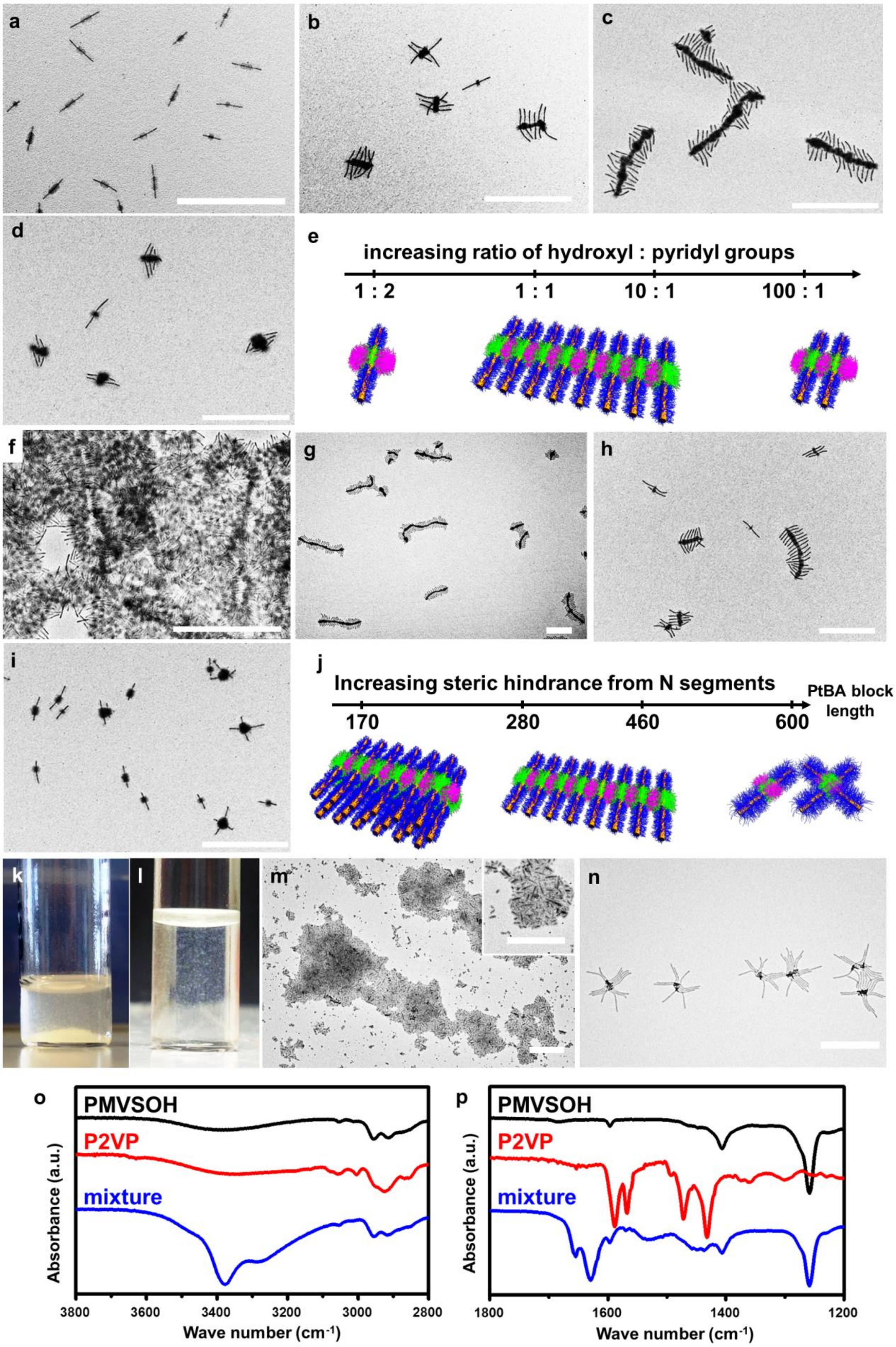

Supplementary Figure 2| Supporting images for the formation of supermicelles from H-bonding interactions. Low resolution TEM image (after solvent evaporation) of supermicelles formed by mixing H_D ($PMVSOH_{105}$) homopolymer with $N\text{-}H_A\text{-}N$

triblock comicelles in *i*-PrOH at a ratio of hydroxyl : pyridyl groups of **a**, 1 : 2; **b**, 1 : 1; **c**, 10 : 1 and **d**, 100 : 1. **e**, Schematic illustration of the supermicelles formed by N-H_A-N triblock comicelles with H_D homopolymer with various ratios of hydroxyl : pyridyl groups. TEM images (after solvent evaporation) of H_D homopolymer ($PMVSOH_{105}$) and N-H_A-N in *i*-PrOH with ratio of hydroxyl : pyridyl groups of 5 : 1, **f**, N = M(PFS_{20}-*b*-$PtBA_{170}$); **g**, N = M(PFS_{20}-*b*-$PtBA_{280}$); **h**, N = M(PFS_{32}-*b*-$PtBA_{460}$); **i**, N = M(PFS_{30}-*b*-$PtBA_{600}$). **j**, Schematic illustration of the supermicelles formed by N-H_A-N triblock comicelles with different PFS-*b*-PtBA diblock copolymers. Photos of the solution of a mixture of **k**, H_D homopolymer and H_A seeds, and **l**, H_D seeds and H_A seeds, both with a mole ratio of hydroxyl : pyridyl groups of 1 : 1, 30 seconds after mixing in *i*-PrOH; **m**, TEM image of the aggregates formed in solution shown in **l** (inset is a zoom-in image of the aggregate). **n,** Low-resolution TEM image (after solvent evaporation) of supermicelles formed by mixing N-H_A-N triblock comicelles in *i*-PrOH with H_D short micelles with the ratio of hydroxyl : pyridyl groups = 1 : 2. **o** and **p**, FTIR spectra of the PMVSOH homopolymer, P2VP homopolymer and a mixture of PMVSOH and P2VP homopolymers (molar ratio of hydroxyl : pyridyl groups of 1 : 1). Scale bars are 1 μm and 500 nm in the inset. Segment H_A = M(PFS_{32}-*b*-$P2VP_{448}$), N = M(PFS_{20}-*b*-$PtBA_{280}$) unless mentioned elsewhere.

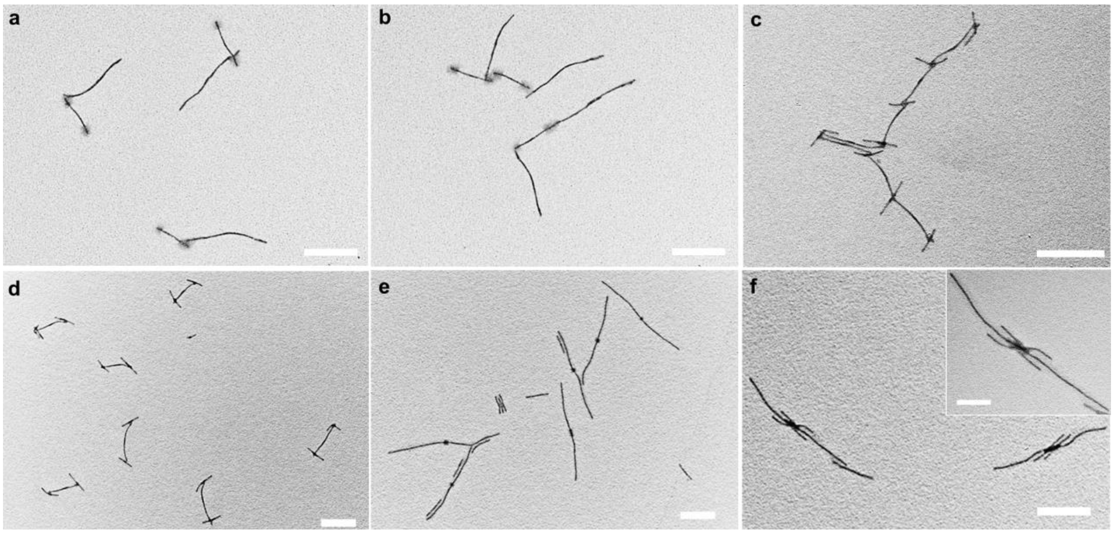

Supplementary Figure 3| Supporting images for the assembly of triblock comicelles via H-bonding. TEM images after solvent evaporation of **a**, dimer and **b**, branched "condensation" supermicelles from triblock comicelles H_D-N-H_D (910 nm) and H_A-N-H_A (380 nm). **c**, "polymeric" supermicelles, and **d**, predominantly "I"-shaped supermicelles from H_D-N-H_D (480 nm) and N-H_A-N (320 nm) triblock comicelles with hydroxyl : pyridyl groups ratios of 3 : 1 and 3.3 : 1, respectively. **e**, the mixture of N-H_D-N (330 nm), and triblock comicelles N-H_A-N (total length 1.6 µm, where the corona-forming P2VP block of the central H_A segment is shorter, see below) triblock comicelles; **f**, the assembled structure of N-H_D-N triblock comicelles (total length 330 nm), and N-H_A-N triblock comicelles (total length 1.5 µm, middle block H_A length 109 nm). All these structures were obtained in *i*-PrOH. Scale bars are 500 nm and that for inset is 200 nm. Segment H_D = M(PFS$_{20}$-*b*-PMVSOH$_{120}$), N = M(PFS$_{20}$-*b*-PtBA$_{280}$), H_A = M(PFS$_{32}$-*b*-P2VP$_{448}$) except that in **e**, H_A = M(PFS$_{25}$-*b*-P2VP$_{250}$).

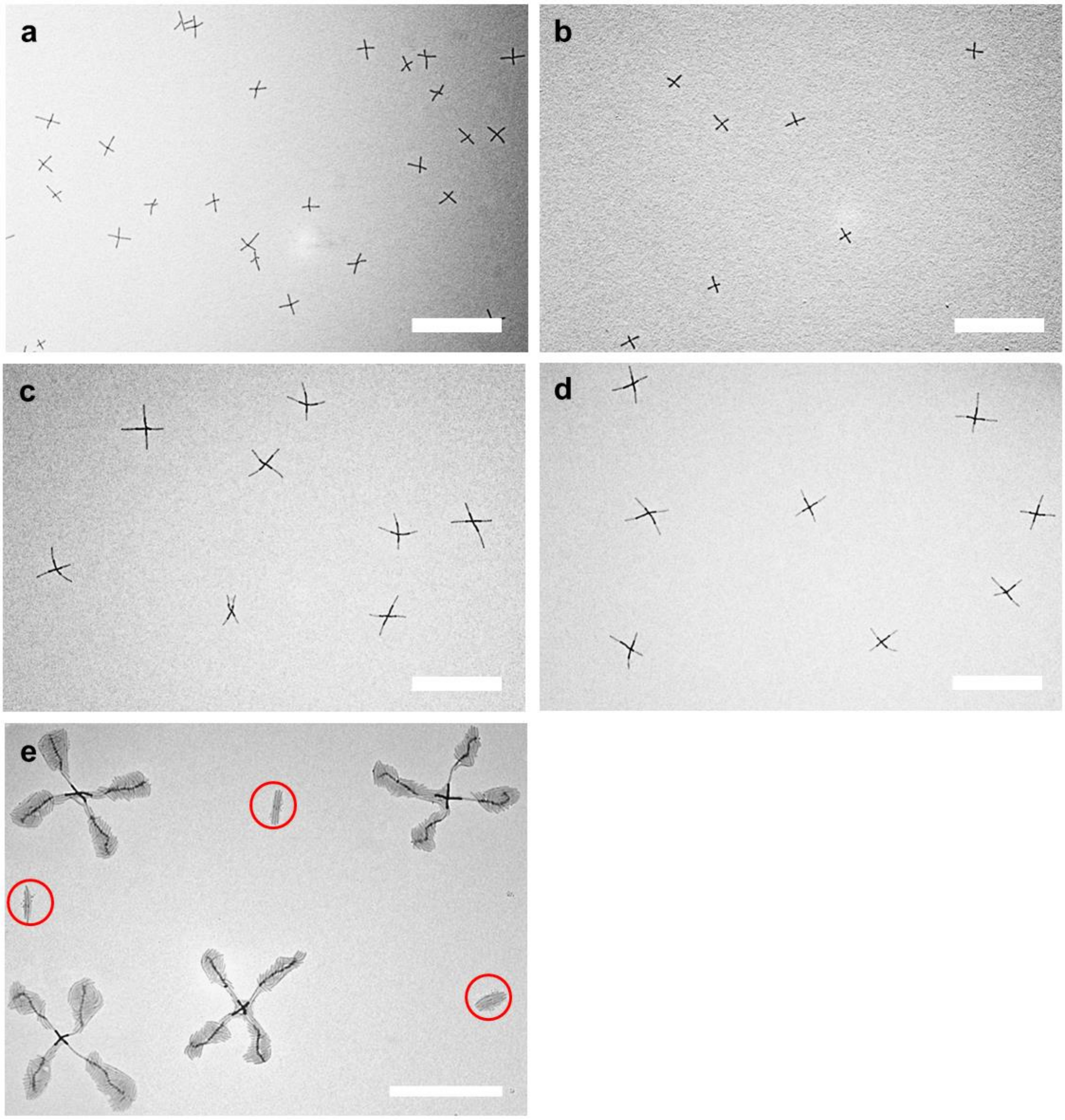

Supplementary Figure 4| Supporting images for the formation of "windmill" supermicelles by hierarchical assembly. Low-resolution TEM images (after solvent evaporation) of the **a**, "cross" supermicelles from the H_A-X-H_A (420 nm) triblock comicelles in MeOH; and the "cross" supermicelles **b**, with crosslinked the X segment (^{XL}X); **c**, after the addition of N segments; **d**, with crosslinked $^{XL}H_A$ segments. **e**, Low-resolution TEM images of the "windmill" supermicelles. Structures marked by the red circles are bundles of individual N-H_D-N triblock comicelles resulting from the H_D seeds that did not become attached to the composite "cross" supermicelles, as shown in Fig. 3f and 3h. All these structures were obtained in *i*-PrOH except the sample shown in image a. Scale bars are 2 μm. Segment H_D = M(PFS_{20}-*b*-$PMVSOH_{120}$), N = M(PFS_{20}-*b*-$PtBA_{280}$), H_A = M(PFS_{25}-*b*-$P2VP_{250}$).

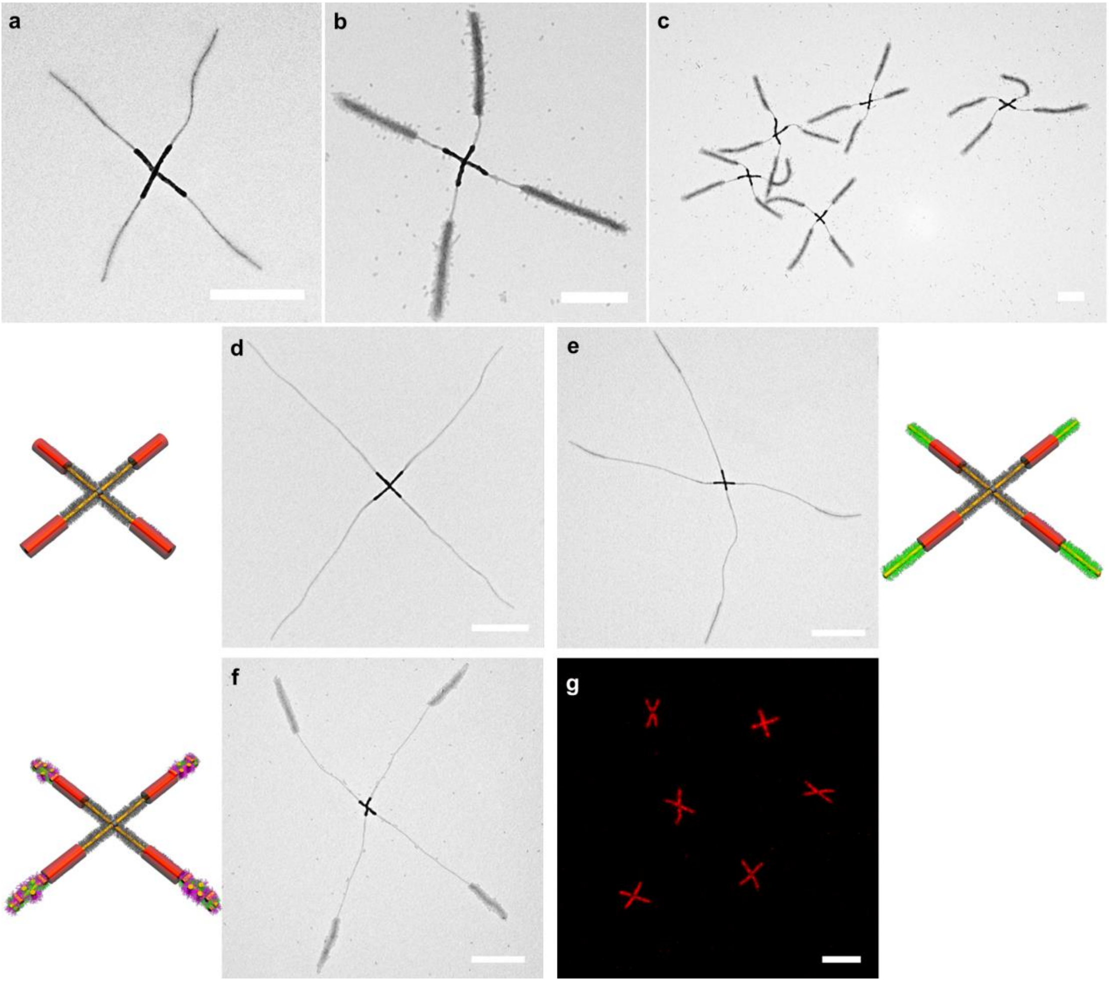

Supplementary Figure 5| Supporting images for the formation of "cross" supermicelles. TEM images of the crosslinked "cross" supermicelles **a**, after the sequential growth of N segments and H_A segments; **b**, after the addition of H_D short micelles (composite "cross" supermicelles). **c**, low-resolution TEM image of the composite "cross" supermicelles. TEM images and their corresponding schematic illustrations of the "cross" supermicelles **d**, with ^{XL}X and $^{XL}H_A$ segments and after the addition of N-red segments (PFS_{20}-*b*-$PtBA_{280}$, end-labeled with the red-dye BODIPY 630/650); **e**, after the addition of another H_A segment; **f**, after the further addition of H_D short micelles (composite "cross" supermicelles). All these TEM images were obtained after the evaporation of solvent (*i*-PrOH). **g**, LSCM image of the composite "cross" supermicelles in *i*-PrOH. Scale bars are 500 nm in **a-c**, 1 μm in **d-f** and 5 μm in **g**. Segment X = M(PFS_{36}-*b*-$PMVS_{324}$), H_D = M(PFS_{20}-*b*-$PMVSOH_{120}$), N = M(PFS_{20}-*b*-$PtBA_{280}$), H_A = M(PFS_{32}-*b*-$P2VP_{448}$), $^{XL}H_A$ = ^{XL}M(PFS_{25}-*b*-$P2VP_{250}$).

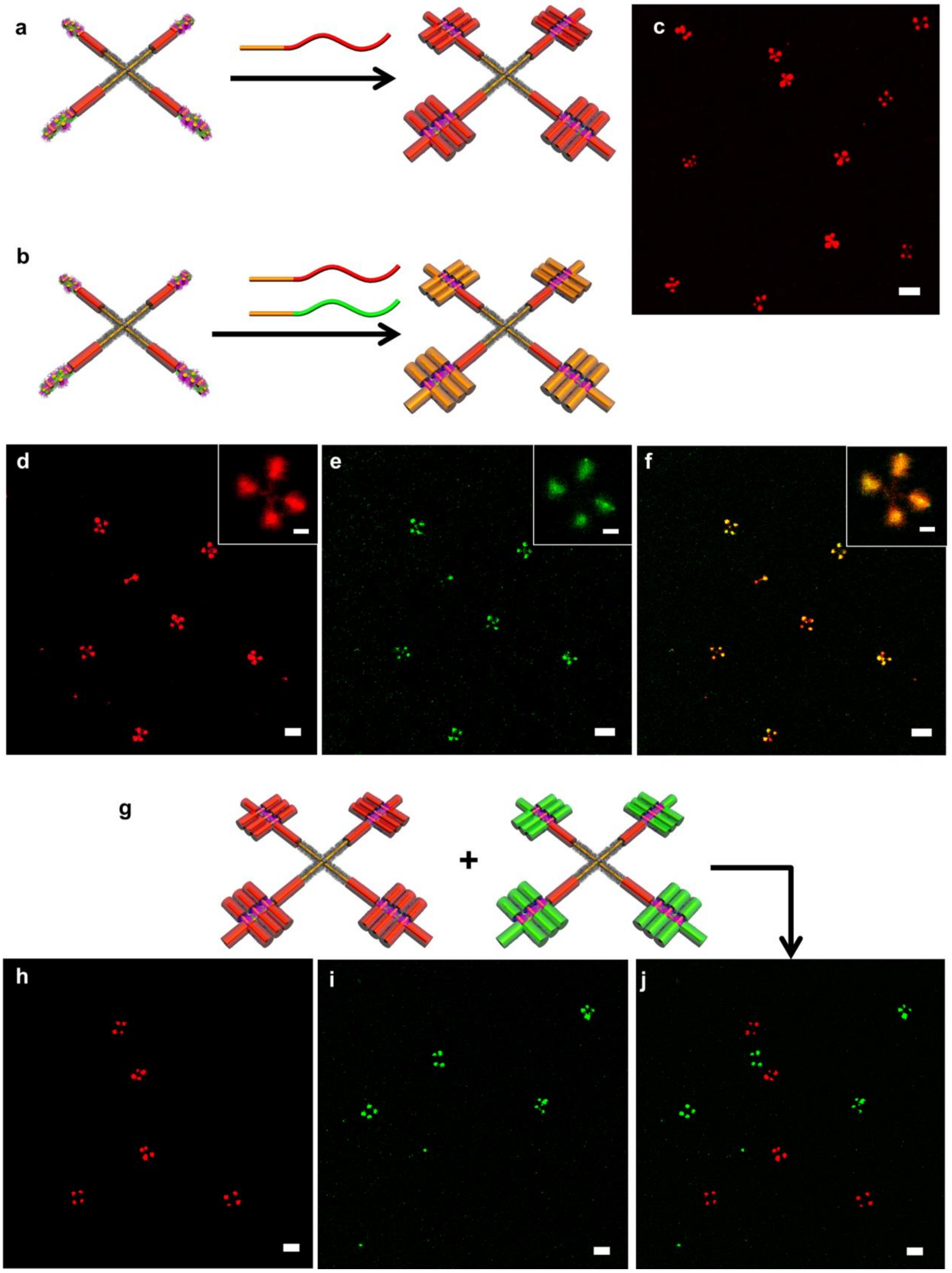

Supplementary Figure 6| Supporting images for fluorescent "windmill"-shaped supermicelles. a, schematic illustration of assembly process of "windmill"-shaped supermicelles labeled with red dye, by adding PFS$_{20}$-*b*-PtBA$_{280}$-red (end-labeled with the red-dye BODIPY 630/650) to composite "cross" supermicelles shown in Supplementary Fig. 5f. **b**, schematic illustration of assembly process for "windmill"-shaped supermicelles labeled with a mixture of red and green dyes, via a similar process but adding a mixture of PFS$_{20}$-*b*-PtBA$_{280}$-red and PFS$_{20}$-*b*-PtBA$_{280}$-green (end-labeled with the green-dye BODIPY FL) with a mole

ratio of 1 : 1. **c**, LSCM image of the "windmill"- shaped supermicelles labeled with red dye. **d-f**, LSCM images of the "windmill"- shaped supermicelles labeled with a mixture of red and green dyes in **d**, red channel, **e**, green channel and **f**, merge channel. **g**, schematic illustration and LSCM images of the mixture of "windmill"- shaped supermicelles labeled with red or green dyes, and **h-j**, LSCM images of this supermicelle mixture (10 days after mixing) : **h**, red channel, **i**, green channel and **j**, merge channel. Inset images in d-f are the high-resolution LSCM images of the "windmill"- shaped supermicelles labeled with a mixture of red and green dyes. All these structures were obtained and characterized in *i*-PrOH. Scale bars are 5 μm in LSCM images and 1 μm in the insets.

Supplementary Table 1| Characteristics of the seed cylinders and the triblock comicelles grown from the seed cylinders.[a]

Segment label and corresponding unimer[b]	central segment length (L_n , nm)	L_w (nm)	L_n (nm)	L_w / L_n [c]	σ [c] (nm)
X, PFS_{36}-*b*-$PMVS_{324}$ (20 nm)	-	23	20	1.16	8
H_A, PFS_{32}-*b*-$P2VP_{448}$ (45 nm)	-	51	45	1.12	16
H_A, PFS_{32}-*b*-$P2VP_{448}$ (109 nm)	-	122	109	1.12	38
H_A, PFS_{32}-*b*-$P2VP_{448}$ (160 nm)	-	179	160	1.12	55
H_A, PFS_{25}-*b*-$P2VP_{250}$ (55 nm)	-	63	55	1.16	22
H_D, PFS_{20}-*b*-$PMVSOH_{120}$ (37 nm)	-	43	37	1.14	14
N, PFS_{20}-*b*-$PtBA_{280}$ (240 nm)	-	280	240	1.16	96
N, PFS_{20}-*b*-$PtBA_{280}$ (370 nm)	-	425	370	1.15	143
N, PFS_{20}-*b*-$PtBA_{280}$ (680 nm)	-	750	680	1.11	225
N-H_D-N, M(PFS_{20}-*b*-$PtBA_{280}$)-*b*-M(PFS_{20}-*b*-$PMVSOH_{120}$)-*b*-M(PFS_{20}-*b*-$PtBA_{280}$) (330 nm)	37	335	330	1.02	45
N-H_D-N, M(PFS_{20}-*b*-$PtBA_{280}$)-*b*-M(PFS_{20}-*b*-$PMVSOH_{120}$)-*b*-M(PFS_{20}-*b*-$PtBA_{280}$) (540 nm)	37	545	540	1.01	55
N-H_A-N, M(PFS_{20}-*b*-$PtBA_{280}$)-*b*-M(PFS_{32}-*b*-$P2VP_{448}$)-*b*-M(PFS_{20}-*b*-$PtBA_{280}$) (320 nm)	45	330	320	1.03	58
N-H_A-N, M(PFS_{20}-*b*-$PtBA_{280}$)-*b*-M(PFS_{32}-*b*-$P2VP_{448}$)-*b*- M(PFS_{20}-*b*-$PtBA_{280}$) (540 nm)	45	560	540	1.03	94
N-H_A-N, M(PFS_{20}-*b*-$PtBA_{280}$)-*b*-M(PFS_{32}-*b*-$P2VP_{448}$)-*b*- M(PFS_{20}-*b*-$PtBA_{280}$) (1.3 µm)	45	1285	1270	1.01	127
N-H_A-N, M(PFS_{20}-*b*-$PtBA_{280}$)-*b*-M(PFS_{32}-*b*-$P2VP_{448}$)-*b*-M(PFS_{20}-*b*-$PtBA_{280}$) (1.5 µm)	109	1550	1450	1.04	290
N-H_A-N, M(PFS_{20}-*b*-$PtBA_{280}$)-*b*-M(PFS_{32}-*b*-$P2VP_{448}$)-*b*-M(PFS_{20}-*b*-$PtBA_{280}$) (1.4 µm)	160	1430	1390	1.03	241
N-H_A-N, M(PFS_{20}-*b*-$PtBA_{280}$)-*b*-M(PFS_{25}-*b*-$P2VP_{250}$)-*b*-M(PFS_{20}-*b*-$PtBA_{280}$) (1.6 µm)	55	1610	1590	1.01	159
H_A-N-H_A, M(PFS_{32}-*b*-$P2VP_{448}$)-*b*-M(PFS_{20}-*b*-$PtBA_{280}$)-*b*-M(PFS_{32}-*b*-$P2VP_{448}$) (380 nm)	240	410	380	1.06	93
H_D-N-H_D, M(PFS_{20}-*b*-$PMVSOH_{120}$)-*b*-M(PFS_{20}-*b*-$PtBA_{280}$)-*b*-M(PFS_{20}-*b*-$PMVSOH_{120}$) (910 nm)	680	950	910	1.05	203
H_D-N-H_D, M(PFS_{20}-*b*-$PMVSOH_{120}$)-*b*-M(PFS_{20}-*b*-$PtBA_{280}$) -*b*-M(PFS_{20}-*b*-$PMVSOH_{120}$) (480 nm)	370	505	480	1.09	144
H_A-X-H_A, M(PFS_{25}-*b*-$P2VP_{250}$)-*b*-M(PFS_{36}-*b*-$PMVS_{324}$)-b-M(PFS_{25}-*b*- $P2VP_{250}$) (420 nm)	20	450	420	1.07	111

a: the lengths of the cylinders were measured from their TEM images and the values were obtained by averaging over 100 readings; b: values in parentheses represent the total length (L_n) of the cylindrical micelles or block comicelles; c: L_n, L_w and σ are the number average micelle length, weight average micelle length, and the standard deviation of the micelle length distribution, respectively, as calculated from the histograms of the length distributions.
